# Spatial transcriptomics reveals gene expression characteristics in invasive micropapillary carcinoma of the breast

**DOI:** 10.1038/s41419-021-04380-6

**Published:** 2021-11-20

**Authors:** Jianke Lv, Qianqian Shi, Yunwei Han, Weidong Li, Hanjiao Liu, Jingyue Zhang, Chen Niu, Guangshen Gao, Yiru Fu, Renyong Zhi, Kailiang Wu, Shuai Li, Feng Gu, Li Fu

**Affiliations:** 1 Department of Breast Cancer Pathology and Research Laboratory, Tianjin Medical University Cancer Institute and Hospital, Tianjin, 300060 China; 2 National Clinical Research Center of Cancer, Tianjin, 300060 China; 3 Key Laboratory of Cancer Prevention and Therapy, Tianjin, 300060 China; 4 Tianjin’s Clinical Research Center for Cancer, Tianjin, 300060 China; 5 Key Laboratory of Breast Cancer Prevention and Therapy, Tianjin Medical University, Ministry of Education, Tianjin, 300060 China

**Keywords:** Breast cancer, Cancer genetics, Tumour heterogeneity

## Abstract

Invasive micropapillary carcinoma (IMPC) is a special histological subtype of breast cancer, featured with extremely high rates of lymphovascular invasion and lymph node metastasis. Based on a previous series of studies, our team proposed the hypothesis of “clustered metastasis of IMPC tumor cells”. However, the transcriptomics characteristics underlying its metastasis are unknown, especially in spatial transcriptomics (ST). In this paper, we perform ST sequencing on four freshly frozen IMPC samples. We draw the transcriptomic maps of IMPC for the first time and reveal its extensive heterogeneity, associated with metabolic reprogramming. We also find that IMPC subpopulations with abnormal metabolism are arranged in different spatial areas, and higher levels of lipid metabolism are observed in all IMPC hierarchical clusters. Moreover, we find that the stromal regions show varieties of gene expression programs, and this difference depends on their distance from IMPC regions. Furthermore, a total of seven IMPC hierarchical clusters of four samples share a common higher expression level of the *SREBF1* gene. Immunohistochemistry results further show that high SREBF1 protein expression is associated with lymph node metastasis and poor survival in IMPC patients. Together, these findings provide a valuable resource for exploring the inter- and intra-tumoral heterogeneity of IMPC and identify a new marker, *SREBF1*, which may facilitate accurate diagnosis and treatment of this disease.

## Introduction

Invasive micropapillary carcinoma (IMPC) is a morphologically distinctive form of breast cancer that has a special inverted growth pattern [1]. It has been reported in multiple organs, including the breast [2], lung [3], colon [4], pancreas [5] and bladder [6], etc. Breast IMPC was first identified by Siriaunkgul and Tavassolil in 1993 [2], and is composed of small, hollow or morula-like clusters of tumor cells without fibrovascular cores, surrounded by clear stromal spaces [7]. Compared with invasive ductal carcinoma-not otherwise specified type (IDC-NOS), IMPC is associated with a higher incidence of lymphovascular invasion and lymph node metastasis and a poorer prognosis [8, 9]. The unique clustered growth pattern and aggressive biological behaviors render IMPC a good model for studying tumor invasion and metastasis. Fu et al. [8] further noted that even if an IMPC tumor was small in diameter or consisted of only a few IMPC proportions, tumor cells were more likely to invade and metastasize. Later, a series of studies proposed the hypothesis of “clustered metastasis of IMPC tumor cells” [10–18]. However, spatial transcriptomics (ST) characteristics have not yet been reported.

ST technology has been designated as the method of 2020 [19]. Spatially resolved transcriptomics [20] combined with microscopic imaging and mRNA sequencing can provide transcriptome data for every spot from different locations in a tissue section. Spatial visualization exploration was realized by analyzing gene expression levels in corresponding tissue regions. Here, we investigated the gene expression characteristics of IMPC cells at the morphological and transcriptomic scales simultaneously using this popular method for the first time.

Therefore, based on a previous series of studies, we employed a spatial transcriptomics sequencing (ST-seq) approach to assess the transcriptome features of IMPC and dissect IMPC tumor heterogeneity in a relatively homogenous population, thus providing in-depth transcriptional information. Our research identifies a potential biomarker of IMPC and explores key genes associated with the progression of IMPC tumors. Thus, this study provides a further basis for precise diagnosis and treatment of IMPC.

## Materials and methods

### Freshly frozen IMPC samples

We collected four freshly frozen IMPC samples, all from breast cancer patients undergoing modified radical mastectomy. Another tissue was collected for the permeabilization optimization. All patients were diagnosed as breast IMPC or IDC-NOS with IMPC components using preoperative core needle biopsy and intraoperative cryosection methods. These patients were diagnosed and treated by the Breast Center of Tianjin Medical University Cancer Institute and Hospital.

We collected the five tissues under the supervision of senior pathologists, slowly washed the tissue blocks with 4 °C PBS, and cut out small pieces of about 6.5 × 6.5 × 5 mm^3^ containing IMPC components. Next, they were embedded in OCT embedding solution (Sakura, #4532, USA), and frozen in isopentane pre-cooled with liquid nitrogen. Cryosections were cut at 4 μm thickness, respectively. Hematoxylin-eosin staining (H&E) (Eosin, Dako CS701, Hematoxylin Dako S3309, bluing buffer CS702) was used to confirm that these tissues contained IMPC once again.

### ST barcoded microarray information

**Fig. 1:**
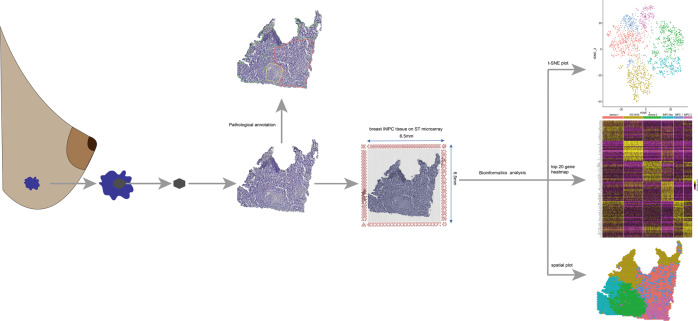
The workflow for spatial transcriptomics (ST) and hierarchical clustering results. Attached a 10 µm thick tissue to the slide for ST sequencing, performed bioinformatics analysis on the sequencing data, and displayed the hierarchical clustering results with t-SNE, heatmap, and spatial graphs.

The ST library preparation microarray (Visium Gene Expression Slide) used in this study was purchased from 10X Genomics (https://www.10xgenomics.com/). A slide includes four capture areas (the size of each area is 6.5 × 6.5 mm), and a total of four tissue sections can be placed. An optimization slide includes eight capture areas, the same size as the gene expression slide. The diameter of each spot on the ST slide is 55 μm. The distance between the centers of the two spots is 100 μm, and a total of 4992 spots cover an area of 6.5 × 6.5 mm^2^ (Fig. 1). Each spot with primers that includes: Illumina TruSeq Read 1 (partial read 1 sequencing primer), 16 nt Spatial Barcode (all primers in a specific spot share the same Spatial Barcode), 12 nt unique molecular identifier (UMI), and 30 nt poly(dT) sequence captures poly-adenylated mRNA for cDNA synthesis. In strict accordance with the ST-seq protocol [21], we used a gene expression slide to perform spatial transcriptome sequencing on four cryosections.

### Tissue Optimization

Cryosections were cut at 10 μm thickness, and placed on the six capture areas on the slide. The other two areas are used for positive and negative controls. The six cryosections on the slide were subjected to permeabilization at different times to capture mRNA, and the reverse transcription was performed to generate fluorescently labeled cDNA. Cheese the permeabilization time that resulted in maximum fluorescence signal with the lowest signal diffusion. Our result found that the best permeabilization time for this experiment was 18 min.

### Cryosection, H&E staining, and brightfield imaging for ST

A −20 °C microtome cryostat (Leica, CM1950) was used to cut tissue cryosections at 10 μm thickness. These sections were mounted onto the gene expression slide. Then the slide was placed on Thermocycler Adaptor with the active surface facing up and incubated 1 min at 37 °C, and fixed for 30 min with methyl alcohol in −20 °C followed by staining with H&E. The brightfield images were taken on a Leica DMI8 whole-slide scanner at 10X resolution. H&E staining cryosections were histologically annotated by three different senior pathologists.

### Permeabilization and reverse transcription

Spatial gene expression was processed using Visum Spatial Gene Expression Slide and Reagent Kit (10x Genomics, PN-1000184). Put the slide on the slide cassette to create leakproof wells for adding reagents. For each well, added 70 µl permeabilization enzyme to make it completely cover every tissue for 18 min. Incubated in the Thermocycler Adaptor at 37 °C for 15 min. Finally, aspirated the tissue permeabilization enzyme with a pipette, and 100 µl 0.1X SSC buffer was added to wash for each well. The poly(dT) sequence of each spot will capture the mRNA released from the cells. Then, 75 μl reverse transcription Master Mix was added for cDNA synthesis. RT Master Mix is usually comprised of Nuclease-free Water, RT Reagent, Template Switch Oligo, Reducing Agent B and RT Enzyme D. After incubating at 53 °C for 45 min, reverse transcription was initiated to generate cDNA with spatial barcode information. Removed the Master Mix reagent, added 75 μl 0.08 M KOH to each tissue section, and incubated at room temperature for 5 min. Removed KOH, added 100 µl Qiagen Buffer EB, then added 75 μl Second Strand Mix (including Second Strand Reagent, Second Strand Primer and Second Strand Enzyme) at 65 °C for 15 min. Finally, removed the above-mentioned reaction reagents, and added 100 μl EB Buffer. After removing the EB Buffer, added 35 μl 0.08 M KOH and incubated at room temperature for 10 min. Transferred 35 µl of cDNA from slide to a tube containing 5 μl Tris (1 M, pH 7.0) for amplification and cDNA library construction.

### cDNA amplification

Used qPCR to confirm the cycle number, according to the following protocol (Supplementary Table S1). Then added 65 μl cDNA Amplification Mix (including Amp Mix and cDNA Primers) to the cDNA sample and put it in the Thermo Fisher Scientific (#4375786). Performed cDNA amplification according to the protocol in Supplementary Table S2. The number of amplification cycles was determined according to the Cq value of qPCR, as follows: 12.2 corresponds to 12 cycles, 13.5 corresponds to 14 cycles, and 15.7 corresponds to 16 cycles. The amplified cDNA was cleaned up, and finally the results were checked for the size of the amplified fragment and the yield of the amplified product. cDNA amplification was performed on an S1000TM Touch Thermal Cycler (Bio Rad).

### Spatial gene expression library construction and sequencing

The spatial gene libraries were constructed using Visum Spatial Library Construction Kit (10X Genomics, PN-1000184). Used chemical methods to break the cDNA into fragments of about 200–300 bp, repaired the ends, added poly(A) tails, and screened the cDNA fragments. Then connected the P7 adapter to the cDNA, introduced the double-ended Index (sample Index) of the sample through PCR amplification, and finally performed fragment screening to obtain the cDNA library. After the library was qualified, 3’ end sequencing was performed using Illumina Novaseq6000 sequencer with a sequencing depth of at least 100,000 reads per spot with pair-end 150 bp (PE150) reading strategy (sequence libraries were generated and sequenced by CapitalBio Technology, Beijing, China).

### Reads alignment

The Spaceranger software was obtained from the 10X Genomics website (https://support.10xgenomics.com/spatial-gene-expression/software/downloads/latest). Reads alignment, filtering, barcode counting, and UMI counting were performed with the Spaceranger count module to generate a feature-barcode matrix using the default and recommended parameters. After the library was constructed, the effective length of Read1 was 28 bp, including 16 bp Barcode and 12 bp UMI. The effective length of Read2 was 91 bp. Read2 contained RNA sequences. Space Ranger uses the STAR algorithm (version 2.5.1b) [22] to map Read2 to Genome Reference Consortium Human Build 38.98 (GRCh38.98).

### Basic ST data analysis

The Spaceranger software was used to perform effective barcode and UMI counts, and generated a gene-barcode expression matrix for each sample, including barcode-labeled spots and gene expression counts. Through the visualized gene number distribution and UMI number distribution figure, all the spots and expressed genes of the sample were evaluated. Imported these data into Seurat 3.2 (R package) for quality control and downstream analysis. Except for certain situations, default parameters were used in all operations. Removed spots with transcripts less than 1000 and mitochondrial transcripts greater than 20%. Sctransform in Seurat was used to normalize UMI count in each spot.

### Hierarchical clustering for each sample

Dimensionality reduction was performed using principal component analysis (PCA) and the first 30 principle components were used to generate clusters by Seurat. Inspired by a graph-based algorithm, we performed Seurat on embedding the spots in the K-nearest neighbor (KNN) [23] graph structure and drew the edges between the spots with similar gene expression patterns. Then tried to decompose the graph into highly interconnected quasi clusters. It first constructed a KNN graph based on Euclidean distance in PCA space. And according to the shared overlap in the Jaccard distance, the edge weight between any two spots was optimized. Two nonlinear dimensionality reduction techniques t-SNE and UMAP were used to visualize the hierarchical clustering results, respectively. The resolution of clustering is 0.8. Next, we used barcodes to generate spatial hierarchical plots. Further, we selected the highly expressed genes with avg_logFC > 0.1 and p_val_adj < 0.05 of every IMPC hierarchical cluster in four samples, and displayed the common highly expressed genes by Venn plot.

### Integrated analysis of multiple samples

Integrated analysis of four samples was carried out by Seurat. Seurat could integrate the gene-barcode expression matrix from multiple runs, normalize those counts into the same sequencing depth and get a new integrated matrix. The steps of hierarchical clustering and visualization were detailed in hierarchical clustering for a single sample.

### Gene functional enrichment analysis

Gene Ontology (GO), Kyoto Encyclopedia of Genes and Genomes (KEGG) and Reactome functional enrichment analyses on the top 50 differentially expressed genes of each hierarchical cluster were performed by ClusterProfiler [24], ReactomePA [25], and DOSE [26], using EnrichProfiler R-packages with Benjamini-Hochberg multiple testing adjustment. The results were visualized using the R package.

### Protein–protein interaction analysis

Protein–protein interaction (PPI) was obtained from human protein interaction data of STRING database [27] with combine_score ≥ 400. The interaction of the top 50 genes of every cluster was extracted from the database. The PPI results were visualized by Cytoscape software, respectively.

### Transcription factor analysis

The transcription factors were predicted within 2000 bp upstream and 500 bp downstream of the transcription start sites for the top 20 genes of each cluster using JASPAR database [28] and the TFBSTools [29]. The gene and TF network for each cluster was visualized using Cytoscape software, respectively. In the figures, we arranged the genes in descending order of degree, and mapped the first 100 combined transcription factors of the gene.

### Gene set enrichment

We considered enrichment in four gene set collections, including GO and KEGG. GSEA was performed by using GSEA software (version 4.0.3) (https://www.gsea-msigdb.org/gsea/index.jsp), which used predefined gene sets from the Molecular Signatures Database (MSigDB v7.1). Gene expression data was calculated by the mean UMI count of gene in one cluster and the rest cluster, respectively. The minimum and maximum criteria for the selection of gene sets from the collection were 0 and 500 genes, respectively. Next, calculated the enrichment score (ES), normalized enrichment score (NES), and false discovery rate (FDR). The FDR value of 25% was selected to determine whether the enriched functional gene set was appropriate.

### Immunohistochemistry staining of FFPE tissue

A total of 132 IMPC and 121 IDC-NOS formalin-fixed and paraffin-embedded (FFPE) tumor tissues were continuously sectioned into 4 μm thickness. Primary antibodies against SREBF1(1:200 dilution, ab191857, Abcam), and FASN (1:3000 dilution, ab128870, Abcam) were used to evaluate protein expression and were reviewed by at least three pathologists. SREBF1 and FASN were graded according to the percentage of positive tumor cells (0 = 0%; 1 < 25%; 2 = 25–50%; 3 > 50%) and the intensity of staining in the tumor (0 = no staining; 1 = weak; 2 = moderate; 3 = high); the two scores were multiplied to obtain an overall score. If the product of the two scores was > 4, they were considered positively stained. And primary antibodies against CD45 (ZM-0183, ZSGB-BIO), CD3 (ZA-0503, ZSGB-BIO), CD8(ZA-0508, ZSGB-BIO), and CD20 (TA800394, ZSGB-BIO) and were also used to independently evaluate protein expression by three pathologists. The immunoreaction was graded as follows: (−) = no positive cells, (+) = 1–25% of the cells stained, (++) = 26–50% of the cells stained and (+++) = 51–100% of the cells stained. The brightfield images were taken on a Leica DMI8 whole-slide scanner at 40X resolution.

### RT-qPCR

Another ten IMPC and nine IDC-NOS tumor samples were collected, and RNA was extracted and reversed, respectively. RT-qPCR was used to detect the expression of *SREBF1* in the IMPC compared with IDC-NOS tumor tissues. *SREBF1* primer is

Forward primer: CGGCGCTGCTGACCGACATC

Reverse primer: CCCTGCCCCACTCCCAGCAT.

*FASN* primer is:

Forward primer: CCATCTACAACATCGACACCA

Reverse primer: CTTCCACACTATGCTCAGGTAG

RT-qPCR was conducted as follows: 95 °C for 30 s, 40 cycles of 95 °C for 5 s and 60 °C for 30 s, then 95 °C for 10 s, 60 °C for 5 s, 95 °C for 5 s. Reaction volume is 10 μl.

### TCGA data analysis

The correlation analysis result was obtained from GEPIA2 website (http://gepia2.cancer-pku.cn/#index). The Pearson test was used for the comparison and *P* value < 0.05 was considered statistically different.

### Statistical analysis

All statistical analyses were performed using R (http://www.r-project.org) and SPSS version 25.0 (SPSS Inc., Chicago, IL, USA). The clinicopathological characteristics were compared using the Pearson *χ*2, Fisher’s exact test and Mann–Whitney *U*-test. Correlations were analyzed using the Spearman or Pearson rank test. Survival analysis (overall survival and disease-free survival) of 82 IMPC and 80 IDC-NOS patients was performed using Kaplan–Meier analysis. Log-rank test, two-sided. *P* < 0.05 was considered to indicate statistical significance.

## Results

### Basic data analysis of transcriptomes

To generate an unbiased hierarchical spatial map of the transcriptome, we collected four freshly frozen samples from IMPC patients (Fig. 1). The clinicopathological information of the four IMPC patients is provided in Supplementary Table S3. The RNA integrity numbers (RIN) of all samples were in the range of 6.67–7.80 and met our requirements (Supplementary Table S4). We acquired transcriptome data for a total of 7242 spots from the four samples. In addition, 10,000 UMIs (median) and 5000 genes (median) per spot were ascertained (Supplementary Table S5). These sequencing data results were consistent with those of other published papers using ST-seq [21, 30, 31]. The numbers of genes and UMIs and the mitochondrial ratio are shown in Supplementary Fig. S1. The spots with a high proportion of mitochondrial genes were filtered out. Next, we demultiplexed the reads and identified their spatial locations in the four tissue samples using location-specific barcodes.

### Spatial genes expression visualization

**Fig. 2:**
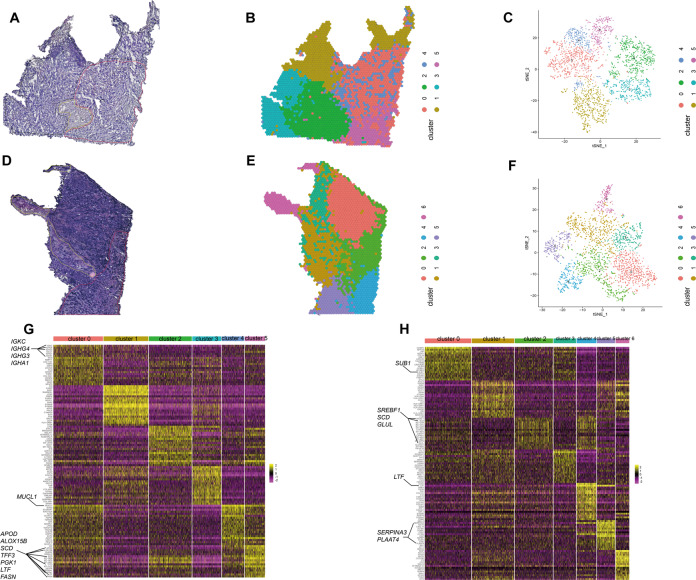
Tumor morphology and hierarchical clustering results. **A** H&E-stained tissue images of sample 1 with marked IMPC (red), IDC-NOS and IMPC-like (black), and stroma (yellow) tissue regions. **B** Hierarchical clustering of the spatial features in sample 1. Each cluster was assigned a color. The cluster 0 (red) and cluster 2 (green) represent interstitial regions. The cluster 1 (yellow) represents IDC-NOS region. The cluster 3 (wathet) is IMPC-like region. Clusters 4 (blue) and 5 (pink) are the IMPC area. **C** t-SNE color visualization of hierarchical clustering profile in sample 1. **D** H&E-stained tissue images of sample 4 with marked IMPC (red), IDC-NOS (black), and stroma (yellow) tissue regions. **E** Hierarchical clustering of the spatial features in sample 4. Each cluster was assigned a color. The cluster 0 (red) and cluster 3 (cyan) represent IDC-NOS regions. The cluster 1 (brown) and cluster 6 (pink) are stromal regions. The cluster 2 (green), 4 (blue), and 5 (purple) are the IMPC area. **F** t-SNE color visualization of hierarchical clustering profile in sample 4. **G**, **H** Heatmap plots of sample 1 and sample 4. t-SNE, t-distributed statistical neighbor embedding.

We applied PCA on highly variable expressed genes across all spots of each sample. Two methods, t-SNE [32] and UMAP [33], were used to visualize the hierarchical clustering results. Next, the clusters were classified into regions using specific barcodes to generate ST maps of IMPC (Fig. 2A–F, samples 1 and 4). The maps of samples 2 and 3 are shown in Supplementary Fig. S2A–F. Different hierarchical clusters were assigned different colors, and the cluster identity was interpolated across the morphology to visualize major spatial patterns within each IMPC sample. The tissue regions were pathologically identified by three senior pathologists. The spatial maps were consistent with the pathological annotations, reflecting the feasibility of distinguishing different spatial regions within a section based on gene expression.

### IMPC clusters were anatomically separate in spatial maps

In sample 1, we identified six main clusters, including cluster 0 and 2 (stromal region), cluster 1 (IDC-NOS region), cluster 3 (IMPC-like region [34], the same as IMPC in morphology), and clusters 4 and 5 (IMPC region) (Fig. 2A, B). The top 20 differentially expressed genes in every cluster are shown in heatmap plots (Fig. 2G).

A total of seven clusters were identified in sample 4 (Fig. 2E). Heatmap plots of the top 20 highly expressed genes are shown in Fig. 2H. Clusters 2, 4, and 5 were located at the IMPC region. Clusters 0 and 3 were identified in the IDC-NOS region. The heatmap plots of samples 2 and 3 are shown in Supplementary Fig. S2G, H. In addition to cancer regions, sample 2 could also distinguish regions of breast ductal epithelial cells (cluster 6) with high expression levels of *PTN*, *RGS2*, *CLU*, and *KRT15* (Supplementary Fig. S2G).

**Fig. 3:**
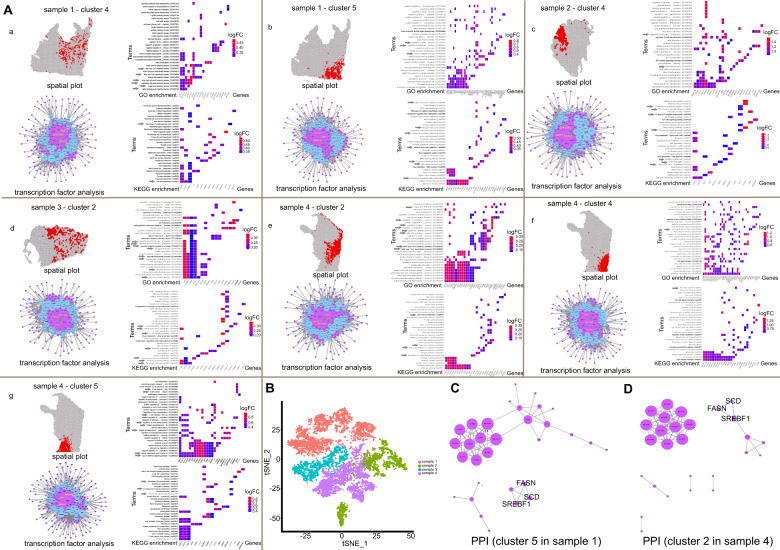
Enrichment, transcription factor analysis and PPI of IMPC cluster in four samples. **A** Highlight spatial hierarchical plot, GO enrichment (biological process, BP), KEGG enrichment and transcription factor analysis among top 50 gene in each IMPC cluster. GO, Gene Ontology. KEGG, Kyoto Encyclopedia of Genes and Genomes. The 30 terms with the lowest p.adjust values of the enrichment results were selected to draw the enrichment plot. The horizontal axis is gene and the vertical axis is term. The color represents the gene’s logFC value. p.adjust: use BH for multiple hypothesis testing, adjusted *P* value. p.adjust < 0.05 is a significant difference. Network diagram of highly expressed genes and transcription factors, the blue dots represent differential genes, and the purple dots represent transcription factors. The larger the node, the more nodes interacted to it. The arrows represent the enrichment terms related to metabolism, tumor immune response, and some important signaling pathways. **B** The t-SNE diagram of the integrated sequencing data. The four samples were completely distinguished. **C, D** The protein–protein interaction (PPI) plots of top 50 highly expressed genes in cluster 5 of sample 1 and cluster 2 of sample 4. The circles are nodes, and the size represents the size of the degree.

Furthermore, we used the JASPAR databases and TFBSTools to predict transcription factors. Several highly expressed genes in IMPC regions were regulated by *ZNF354C*, *SOX10*, and *MZF1* (Fig. 3A). These three transcription factors have been reported to be related to tumorigenesis and progression [35–37] and may be the key factors in the progression of IMPC.

IMPC tumor regions in samples 1 and 4 were composed of multiple hierarchical clusters. Different IMPC clusters exhibited the characteristics of anatomical separation, and were grouped together rather than intermixed (Fig. 2B, E and Supplementary Fig. S2B, E). In contrast, IMPC regions in samples 2 and 3 were composed of only one cluster. Subsequently, we integrated the sequencing data of four samples to generate a merged gene-barcode expression matrix for PCA and visualization (Fig. 3B). The four samples were also clearly separated in the t-SNE plot, further demonstrating extensive heterogeneity between different samples.

This study first delineated the spatial transcriptome of IMPC, visually displaying the gene expression diversity in IMPC regions, and also revealed inter- and intra-tumoral heterogeneity and uncovered the unexplored landscape of the IMPC tumor region, where multiple tumor subclones converge in different spatial regions.

### Genetic heterogeneity was related to metabolic reprogramming in IMPC

Two clusters represented two different metabolic states in the IMPC region of sample 1. Sample 1 has six hierarchical clusters, two of which are IMPC clusters (i.e., clusters 4 and 5). The enrichment analyses showed that the top 50 up-regulated genes in cluster 4 of sample 1 were mainly enriched for lipid metabolism, such as unsaturated fatty acid metabolic, and long-chain fatty acid metabolic (Fig. 3A-a). GSEA showed a strong association with fatty acid beta oxidation and cilium assembly, etc (Supplementary Fig. S3A). We observed higher expression of *SCP2* in cluster 4 than in the other clusters, which was associated with lipid metabolism (Fig. 2G). Cluster 4 was an enhanced lipid metabolism group.

The functional enrichment results also showed that cluster 5 was associated with lipid metabolism and carbohydrate metabolism, including biosynthesis of unsaturated fatty acids, and glycolysis/gluconeogenesis (Fig. 3A-b). High levels of lipid metabolism-related genes (e.g., *APOD*, *ALOX15B*, *SCD*, *LTF*, and *FASN*) and carbohydrate metabolism-related genes (e.g., *TFF3* and *PGK1*) were found in cluster 5 (Fig. 2G). Among the top 50 differentially expressed genes of cluster 5 in sample 1 and cluster 2 in sample 4, a protein interaction relationship was identified among SREBF1, FASN, and SCD (Fig. 3C, D).

In contrast, the enrichment results of cluster 1(IDC-NOS region) showed correlations in the tumor immune response and some other signaling pathways (Supplementary Fig. S4A). Cluster 3 (IMPC-like region) was a special histology area, and the enrichment results of the top 50 up-regulated genes were enriched for the tumor immune response, cell-substrate adhesion and some signaling pathways (Supplementary Fig. S3B), similar to cluster 1.

Only one IMPC cluster represented one metabolic state in sample 2 or sample 3. Sample 2 has seven hierarchical clusters. A total of five clusters were identified in sample 3. However, in sample 2 or sample 3, only one cluster of metabolic enhancement was found in the IMPC region. Notably, the top 50 differentially expressed genes for cluster 4 in sample 2 were enriched for lipid metabolism, amino acid metabolism, cilium, and the tumor immune response (Fig. 3A-c and Supplementary Fig. S3C). Specifically, highly expressed genes of cluster 4 in sample 2 were enriched for microvillus. We observed increased expression of lipid metabolism-related genes *PLPP5*, *XBP1*, and *ESR1* and amino acid metabolism-related gene *GATA3* in cluster 4 of sample 2 (Supplementary Fig. S2G).

The differentially expressed genes for cluster 2 in sample 3 were enriched for lipid metabolism and carbohydrate metabolism (Fig. 3A-d). In sample 3, the IMPC region (cluster 2) was highly expressed in *ADAMTS1*, a gene related to lipid metabolism, and *PFKFB3*, *ENOSF1*, and *GPI*, which are related to carbohydrate metabolism (Fig. S2H). The enrichment results of highly expressed genes in the IDC-NOS region (cluster 0 of sample 3) were related to signaling pathways and immune responses (Supplementary Fig. S4B).

Three IMPC clusters represented three different metabolic states in sample 4. Sample 4 has seven hierarchical clusters, three of which are IMPC clusters. Furthermore, sample 4 was characterized by obvious heterogeneity within the IMPC region, including clusters 2, 4, and 5. In cluster 2, the functional enrichment analyses showed that the top 50 genes were correlated with lipid metabolism, amino acid metabolism, cilium assembly and microvillus (Fig. 3A-e and Supplementary Fig. S3D). Higher expression levels of lipid metabolism-related genes (*SREBF1* and *SCD*) and amino acid metabolism-related genes (*GLUL*) were observed in cluster 2 (Fig. 2H).

We performed enrichment analysis on cluster 4. Lipid metabolism was found to be the main term (Fig. 3A-f and Supplementary Fig. S3E), which was further supported by high expression of the lipid metabolism gene *LTF* (Fig. 2H). Genes for cluster 5 were enriched for lipid metabolism, carbohydrate metabolism and immune response terms (Fig. 3A-g and Supplementary Fig. S3F). We observed higher expression levels of some genes in cluster 5 (Fig. 2H), such as *SERPINA3*, *LTF*, *PLAAT4* (lipid metabolism), and *ALDOA* (carbohydrate metabolism).

IDC-NOS regions (cluster 0 and 3) were dominated by enriched terms related to multiple metabolisms, immune response, and other cancer signaling pathways (Supplementary Fig. S4C, D). Our results elucidated that the immune response term was enriched in all IDC-NOS clusters. Next, 50 IMPC and 41 IDC-NOS FFPE tumor tissues were used for immunohistochemical staining of CD45, CD3, CD8, and CD20. We found significantly higher expression levels of CD45, CD3, and CD8 in the IDC-NOS group than in the IMPC group (Mann–Whitney *U*-test, all *P* < 0.05), but no difference in CD20 was observed (*P* > 0.05) (Supplementary Table S6). And T lymphocytes were more numerous in IMPC and IDC-NOS tumors than B lymphocytes (Mann–Whitney *U*-test, both *P* < 0.01, Supplementary Table S7). It suggests that IDC-NOS tumors have noticeable numbers of tumor infiltrating lymphocytes representing the immune response.

Polyclonal samples 1 and 4 originated from pN1 and pN2 patients with lymphovascular invasion, respectively. In contrast, patients 2 and 3 had no lymph node metastasis and no lymphovascular invasion (Supplementary Table S3). Extensive intra-tumor heterogeneity correlates with a higher N stage and a higher rate of lymphovascular invasion.

### Spatial visualization characteristics of the tumor microenvironment

**Fig. 4:**
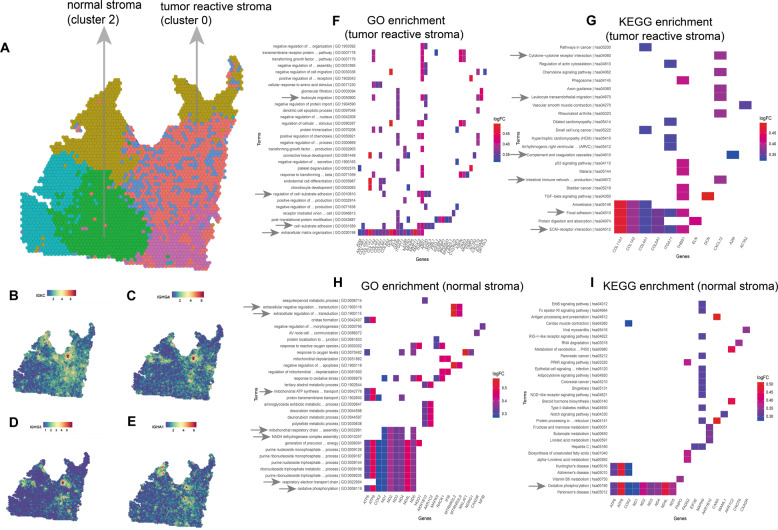
The spatial distribution of stromal regions affects the gene expression of IMPC regions in sample 1. **A** The spatial distribution position of two stromal clusters (cluster 0 and 2). The stroma (green cluster) is far from IMPC regions (blue and pink clusters). Another stroma (red cluster) is near IMPC regions. **B–E** The expression level of *IGKC*, *IGHG4, IGHG3*, and *IGHA1* genes on the spatial plots. **F, G** The enrichment results of cluster 0 highly expressed top 50 genes on GO (BP) and KEGG. **H, I** The enrichment results of cluster 2 highly expressed top 50 genes on GO (BP), and KEGG. The arrows represent that these terms were linked to immune response, cellular interaction, complement pathway, and oxidative phosphorylation, etc.

In sample 1, the stromal region has two clusters, namely, cluster 0 and cluster 2. In the spatial map, cluster 0 was close to the IMPC region, and cluster 2 was farther away from the IMPC region (Fig. 4A). *IGKC*, *IGHG4, IGHG3,* and *IGHA1* were highly expressed in cluster 0 (Fig. 4B–E). Functional enrichment analysis of cluster 0 showed that it was associated with cellular interaction, complement pathway and immune response (Fig. 4F, G and Supplementary Fig. S5A–H). The complement system plays a major and complex role in the tumor microenvironment. For one thing, the complement system was found to be related to immune surveillance for antitumor [21, 38]. For another thing, some studies have reported that the complement system was distinctly important for promoting tumor growth, which has an immunosuppressive effect in tumor immunity [39, 40]. Our study revealed that the complement pathways was enriched in the tumor reactive stromal region (cluster 0), which played a vital role in the growth of IMPC cells.

In contrast, genes for cluster 2 were enriched for oxidative phosphorylation, extracelluar and intercellular transport terms (Fig. 4H, I and Supplementary Fig. S5I). Although both are stromal clusters, their enrichment terms are totally different. In particular, the terms interstitial support, transduction and tumor immune response were mainly enriched in cluster 0 (close to the IMPC region in spatial position), while cluster 2 (far away from the IMPC area) was not. In addition, only one metabolic enhancement occurred when many interstitial cells were mixed in the IMPC region (e.g., cluster 4). However, when fewer mesenchymal cells were present (such as cluster 5), two types of metabolic enhancement occurred simultaneously. Therefore, the difference in spatial distribution between the stroma and the tumor may affect the immune response of stromal region. The metabolic mode of IMPC also changes when more mesenchymal cells are close to the IMPC region.

### High IMPC-specific *SREBF1* expression was associated with malignant biological behavior

**Fig. 5:**
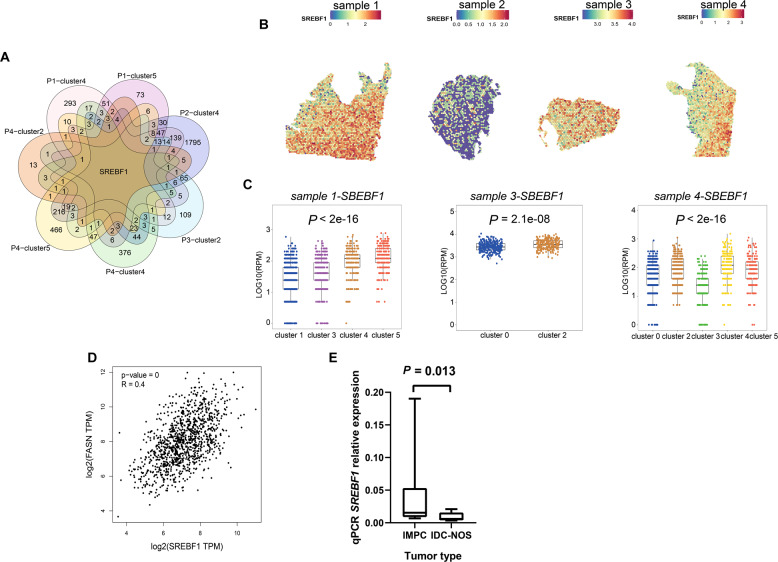
*SREBF1* was a common highly expressed gene in the IMPC clusters of four samples. **A** The Venn plot showed that *SREBF1* was a common high expression gene in all IMPC clusters of the 4 samples. **B** *SREBF1* was highly expressed in IMPC clusters of each sample in spatial plots. **C** Boxplot of *SREBF1* expression level in IMPC clusters versus IDC-NOS clusters. Bar represents median, and boxplot represents quartiles; scale in log10(RPM). Student’s *t*-test for comparison between two groups in each sample. The difference was significant in sample 1 (*P* < 2e-16), sample 3 (*P* = 2.1e-08) and sample 4 (*P* < 2e-16). **D** The correlation analysis between *SREBF1* and *FASN* using TCGA database. The Pearson correlation coefficient was used, two-sided. *P* < 0.05 was considered to indicate statistical significance. **E** *SREBF1* was more highly expressed in IMPC tumor tissues using RT-qPCR. Bar represents median, and boxplot represents quartiles. Student’s *t*-test for comparison. The two groups were significantly different (*P* = 0.013).

Excitingly, *SREBF1* was a common highly expressed gene in IMPC clusters of all samples (“Methods”, Fig. 5A, B) and was the only gene that was highly expressed in all IMPC clusters. Furthermore, the expression level of *SREBF1* in IMPC clusters was significantly higher than that in IDC-NOS clusters (Student’s *t*-test, *P* < 0.05) (Fig. 5C).

*FASN* is a target gene of *SREBF1* [41], and a positive correlation was found between them (*R* = 0.400, *P* = 0.000) using the TCGA database (Fig. 5D). In addition, in IMPC clusters of samples 1 and 4, the expression levels of *FASN* were significantly higher than those in IDC-NOS (Student’s *t*-test, *P* < 0.05) (Supplementary Fig. S6A–G).

**Fig. 6:**
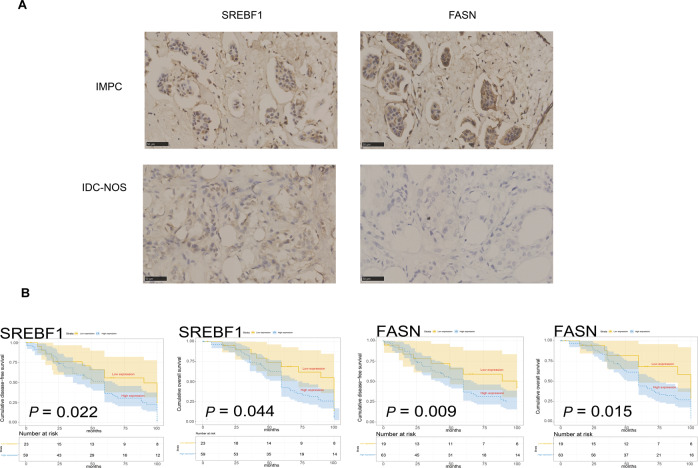
Clinical outcomes associated with the expression of the SREBF1 and FASN proteins in 82 patients with IMPC and 80 with IDC-NOS. **A** Images of immunohistochemical staining for SREBF1 and FASN in patients with IMPC (*n* = 82) and IDC-NOS (*n* = 80). The upper panel shows high expression of SREBF1 and FASN in patients with IMPC; the lower panel shows low expression of the SREBF1 and FASN proteins in patients with IDC-NOS, x400 magnification. **B** The association of SREBF1 and FASN protein expression with disease-free survival (DFS) and overall survival (OS). The light panel shows SREBF1, and the right panel shows FASN, with patients with IMPC stratified by high and low protein expression. Log-rank test, two-sided. The *P* values for DFS of SREBF1 and FASN were 0.022, and 0.009, respectively; the *P* values for OS of SREBF1 and FASN were 0.044 and 0.015, respectively. *P* < 0.05 was considered to indicate statistical significance.

The RT-qPCR results revealed that *SREBF1* and *FASN* showed significantly high expression in ten IMPC and nine IDC-NOS tumor tissues, respectively (Student’s *t*-test, *P* = 0.013 and 0.037) (Fig. 5E and Supplementary Fig. S6H). In all, 82 IMPC and 80 IDC-NOS FFPE tissues were used for immunohistochemical staining of SREBF1 and FASN. The expression of SREBF1 and FASN was significantly high in IMPC, and the difference was statistically significant (Pearson *χ*2 test, *P* = 0.000) (Fig. 6A and Supplementary Fig. S7A). The expression of SREBF1 and FASN proteins was significantly associated with a higher level of lymph node metastasis in IMPC (SREBF1, *R* = 0.405, *P* = 0.000; FASN, *R* = 0.521, *P* = 0.000) (Supplementary Fig. S7B). The high expression levels of the SREBF1 and FASN proteins indicated a poor prognosis for overall survival and disease-free survival time (based on the log-rank test, *P* < 0.05 for both) (Fig. 6B).

## Discussion

Breast cancer accounts for 24.5% of the total number of new cancer cases in women worldwide, overtaking lung cancer as the most common cancer in the world [42]. IMPC is classified as a special histologic subtype of breast cancer. The unique growth pattern and histological morphology caused by polarity reversal, facilitate IMPC tumor cell clusters’ detachment, and these clusters break into single cells or smaller clusters, thereby promoting invasion and metastasis [10]. To explore the inter- and intra-tumoral heterogeneity of IMPC and understand why IMPCs have aggressive biological behaviors, such as very high rates of lymph vascular invasion and lymph node metastasis [8, 11], we conducted this study to investigate the gene expression characteristics of IMPC at the spatial transcriptome level.

Single-cell RNA-seq can identify oncogenic drivers, explore the heterogeneity and reconstruction of evolutionary lineages [43–45]. Although breakthroughs in the immune treatment and chemoresistance of various malignant tumors, such as breast cancer [46] and hepatocellular carcinoma [47], have been achieved, information on the spatial locations of tumor tissues is lacking. In our study, we applied ST-seq, which quantified an array of transcriptomes across a tissue section, to unravel gene spatial expression characteristics in IMPC.

Some studies have reported that the tumor heterogeneity of breast cancer is correlated with changes in chromosomes and genes [48]. Heterogeneity affects multiple developmental stages of tumor. Nicholas et al. [49] observed that tumor subpopulations may be anatomically separate or intermixed. That is, a “tumor subclone” can be separated in different areas, or scattered and crossed in the same region. Studying the distribution characteristics of tumors subclones enables us to determine the developmental organization of tumor growth and the migratory pattern. In our study, two of the four samples had different clusters in the IMPC region, and “subclones” were clustered in different regions and separated from each other. The patterns of special growth and development characterized by IMPC subpopulations require further research and analysis.

Several studies have found that tumor cells can re-adapt to the environment through metabolic reprogramming to maintain advantages in proliferation and metastasis [50, 51]. All IMPC clusters had abnormal lipid metabolism enhancement. Fatty acids are the main energy substances of the body in the tumor-bearing state, and may support the growth of tumor cells by providing metabolic substrates for energy production [52]. Under the energy stress of tumor cells, the fatty acid β oxidation pathway can also provide energy for tumor cells. This form may be an important metabolic change in tumor development [53]. Abnormal carbohydrate metabolism enhancement is also a characteristic change in IMPC. The Warburg effect [51] holds that glycolysis in tumor cells is abnormally active, and is preferentially used to provide cells with energy. The citric acid cycle (TCA) is the key means for prostate cells to transform into malignant tumor cells [54]. Some researchers have found that the fatty acid-β oxidation pathway is enhanced in prostate cancer and provides ATP and acetyl-CoA, which can also accelerate the oxidative metabolism of citric acid [53]. Metabolic reprogramming is an important feature that distinguishes IMPC and IDC-NOS and may also be a key mechanism leading to the special growth mode and high rates of invasion and metastasis.

Highly expressed genes in the IDC-NOS regions of the four samples were mainly enriched in the immune response of the tumor. The immunohistochemistry results also confirmed the presence of more lymphocyte infiltration in IDC-NOS regions than in IMPC regions. The IMPC region lacks the immune-surveillance antitumor effect of T lymphocyte infiltration [55]. A fierce interaction occurs between immune cells and tumor cells in IDC-NOS regions. Tumor cells interact with the immune microenvironment, and immune cells play an important role in tumor immunity, indirectly or directly inhibiting the growth and invasion of tumor cells. In addition, the highly expressed genes of IDC-NOS were also related to the formation of extracellular matrix and cell-cell interaction. Reports in the literature indicate that the extracellular matrix of breast cancer stroma is remodeled, which plays an important role in the invasion and metastasis of tumor cells [56]. Taken together, the IDC region has a stronger tumor immune response and more lymphocyte infiltration than the IMPC. The lack of an antitumor effect of lymphocyte infiltration may be one of the reasons for the malignant biological behavior of IMPC.

In our study, a new gene expression characterization of the stroma in the proximity of IMPC was elucidated by spatial mapping. The stromal regions show different gene expression characteristics, which are related to the distance between them and IMPC. This study revealed high levels of immunoglobulin-related genes within the stroma close to IMPC, whereas the stroma far away from IMPC was related to oxidative phosphorylation. A high level of immunoglobulin secretion indicates that the immune effect of lymphocytes in the cancer area is active. This type of stroma is regarded as an emerging hallmark of tumor invasion and metastasis [21]. The phenomenon of different interstitial functions caused by different spatial distributions can be clearly observed by spatial atlas gene expression.

Microvilli are abundant on the outer surfaces of the IMPC tumor cell clusters under electron microscopy, and most of the microvilli are gathered in the movement direction of the IMPC cell clusters [10]. The assembly and formation of cilia can significantly promote the invasion and metastasis of pancreatic cancer, lung cancer, lymphoma and other tumors [57].

*SREBF1* is a key transcription factor regulating *FASN* in lipid metabolism [58] and is regulated by the PI3K-mTORC1-AKT signaling pathway [59]. Phosphorylation of mTORC1-dependent phospholipid-1 can promote *SREBF1* to enter the nucleus [60]. *FASN* is a key enzyme for the de novo synthesis of long-chain fatty acids. Under pathological conditions, *SREBF1* and *FASN* have been reported to be a pair of metabolically related oncogenes [58], which have been confirmed to be related to prostate cancer and breast cancer [61–64]. In recent years, antitumor treatments targeting enzymes or transcription factors in cell metabolism have become a research hot spot. *SREBF1/FASN* may play an extremely important role in the invasion and metastasis of IMPC. The specific mechanism and whether they can be used as target genes for IMPC precision therapy require further research and verification.

In summary, this study reports ST maps of IMPC for the first time and further improves the research hypothesis of “clustered metastasis of IMPC tumor cells” postulated in our previous research. Furthermore, we revealed that IMPC tumor cells have extensive heterogeneity, closely related to metabolic reprogramming based on lipid metabolism. *SREBF1* is associated with the aggressive biological behavior of IMPC. These results provide a powerful basis and targeted molecules for the precise diagnosis and treatment of IMPC.

## Supplementary information

Figure S1

Figure S2

Figure S3

Figure S4

Figure S5

Figure S6

Figure S7

Supplementary figure legends

Table S1

Table S2

Table S3

Table S4

Table S5

Table S6

Table S7

Checklist

## Data Availability

Sequencing data are deposited at the Genome Sequence Archive (GSA), under the accession number HRA001442.

## Code availability

R and other custom scripts used to analyze the data are available upon request.
