# Supplementary figures and images for: Spatial transcriptomics reveals gene expression characteristics in invasive micropapillary carcinoma of the breast
Source: Cell Death Dis. 2021 Nov 20;12(12):1095. doi: 10.1038/s41419-021-04380-6 (PMC8605000; doi:10.1038/s41419-021-04380-6)

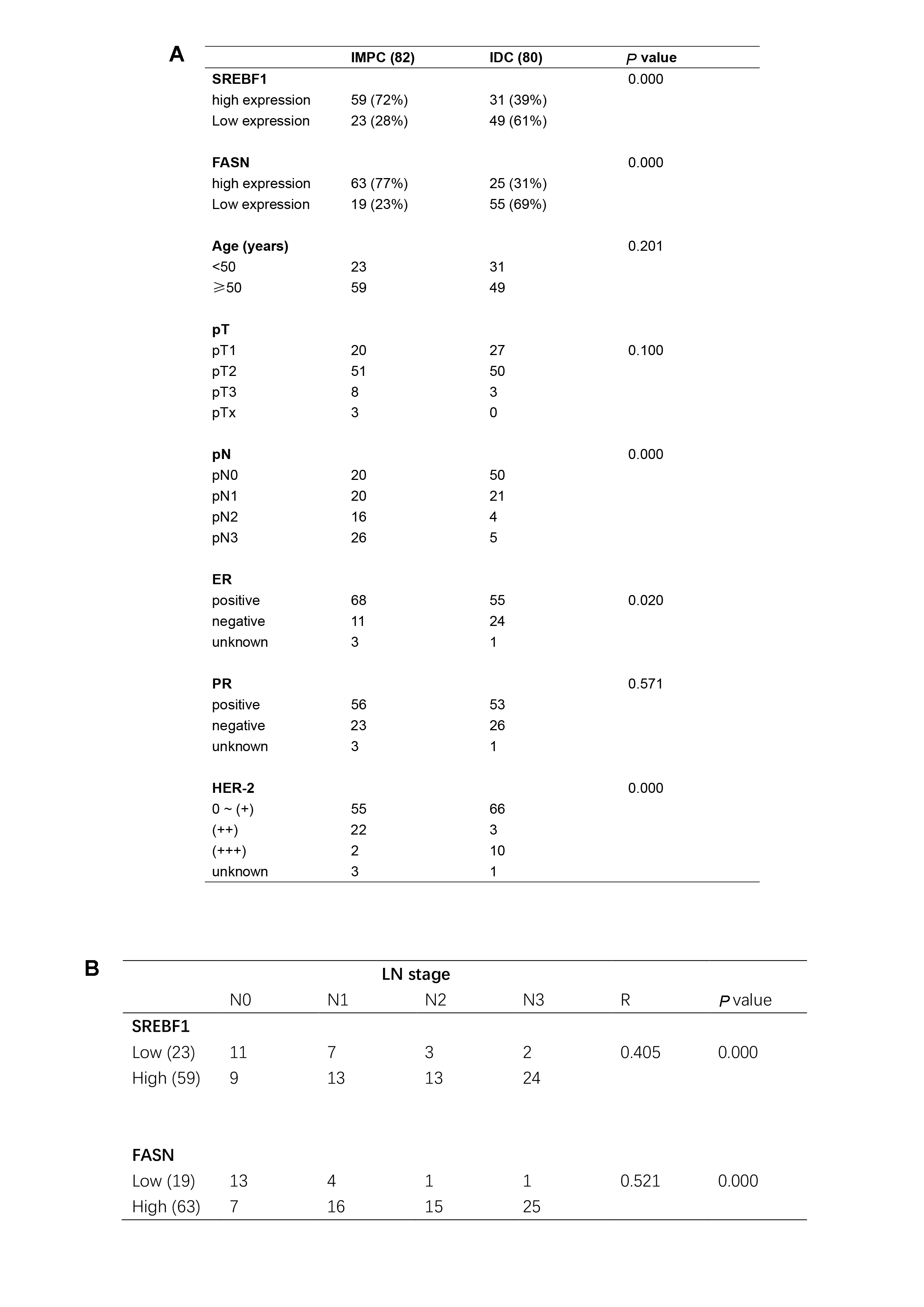

Supplement: Supplementary file 7 — Figure S7 [file 41419_2021_4380_MOESM7_ESM.tif]
