# Supplementary material for: Spatial transcriptomics reveals gene expression characteristics in invasive micropapillary carcinoma of the breast
Source: Cell Death Dis. 2021 Nov 20;12(12):1095. doi: 10.1038/s41419-021-04380-6 (PMC8605000; doi:10.1038/s41419-021-04380-6)
Supplement: Supplementary file 8 — Supplementary figure legends [file 41419_2021_4380_MOESM8_ESM.docx]

**Supplementary figure legends**

**Figure S1. Distribution of genes number, UMIs number and mitochondrial genome of all spots in the 4 samples, respectively. A** The violin plots of the numbers of genes, UMIs and mitochondrial gene ratio. **B** The spatial distribution profile of genes and UMIs number.

**Figure S2. Tumor morphology and hierarchical clustering results. A** H&E-stained tissue images of sample 2 with marked IMPC (red) and stroma (yellow) tissue regions. **B** Hierarchical clustering of the spatial features in sample 2. Each cluster was assigned a color. The cluster 4 (blue) is IMPC region. Cluster 6 (pink) is the mammary epithelial region. **C** t-SNE color visualization of hierarchical clustering profile in sample 4. **D** H&E-stained tissue images of sample 3 with marked IMPC (red), IDC-NOS (black), and stroma (yellow) tissue regions. **E** Hierarchical clustering of the spatial features in sample 3. Each cluster was assigned a color. The cluster 0 (red) represent IDC-NOS regions. The cluster 2 (green) is the IMPC region. **F** t-SNE color visualization of hierarchical clustering profile in sample 3. **G-H** Heatmap plots of sample 2 and sample 3.

**Figure S3. GO (cell component, CC) and GSEA enrichment analysis. A** The GSEA enrichment of sample 1 - cluster 4 showed a strong association with lipid metabolism and intraciliary transport involved in cilium assembly. **B** The GO enrichment results of the top 50 up-regulated genes in sample 1 - cluster 3. The arrows indicated that the terms were related to tumor immune response, cell-substrate adhesion and signaling pathways. **C** The GO enrichment of sample 2 - cluster 4. The arrows represent that the terms were associated with cilium and microvillus. **D** The GSEA enrichment of sample 4 - cluster 2. The arrows represent that the terms were associated with cilium and microvillus. **E and F** The GSEA enrichment of sample 4 - cluster 4 and 5. The arrows represent that the terms were associated with lipid metabolism.

**Figure S4. Enrichment results of IDC-NOS cluster in a total of 4 samples. A** The highlight spatial hierarchical plot and the enrichment results of cluster 1 highly expressed top 50 genes on GO (BP) and KEGG in sample 1. The arrows indicated that the terms of enrichment were associated with tumor immune response and some other signaling pathways. **B** The highlight spatial hierarchical plot and the enrichment results of cluster 0 highly expressed top 50 genes on GO (BP) and KEGG in sample 3. The arrows represent that the terms of enrichment were also associated with signaling pathways, and immune responses. **C and D** The highlight spatial hierarchical plot and the enrichment results of cluster 0 and 3 highly expressed top 50 genes on GO (BP) and KEGG in sample 4. The arrows indicated that enriched terms were related to mammary gland development, multiple metabolisms, immune response, and other cancer signaling pathways.

**Figure S5. GSEA enrichment analysis of cluster 0 & 2 in sample 1, cluster 2, 4 & 5 in sample 4.**

**Figure S6. *FASN* was highly expressed gene in the IMPC clusters. A** *FASN* was highly expressed in IMPC clusters of each sample in spatial plots. **B** Boxplot of *FASN* expression level in IMPC clusters versus IDC-NOS clusters. The difference was significant in sample 1 (*P* < 2e-16), sample 3 (*P* = 0.055) and sample 4 (*P* = 6.1e-15). **C** *FASN* was more highly expressed in IMPC tumor tissues using RT–qPCR. Bar represents median, and boxplot represents quartiles. Student’s t-test for comparison. The two groups were significantly different (*P* = 0.037).

**Figure S7 The SREBF1 and FASN protein expression levels of 82 IMPC and 80 IDC-NOS patients. A** The expression of SREBF1 and FASN was significantly high in IMPC patients. *P* values were calculated for Pearson χ2 and Fisher’s exact test, two-sided. **B** The correlation between the expression of SREBF1 and FASN proteins and IMPC lymph metastatic stage. The Spearman correlation coefficient was used, two-sided. *P* < 0.05 was considered to indicate statistical significance. LN stage: lymph node stage.
